# Variable climate suitability for wheat blast (*Magnaporthe oryzae* pathotype Triticum) in Asia: results from a continental-scale modeling approach

**DOI:** 10.1007/s00484-022-02352-9

**Published:** 2022-08-22

**Authors:** Carlo Montes, Sk. Ghulam Hussain, Timothy J. Krupnik

**Affiliations:** 1 International Maize and Wheat Improvement Center (CIMMYT), Texcoco, Mexico; 2 International Maize and Wheat Improvement Center (CIMMYT), Dhaka, Bangladesh

**Keywords:** Crop damage, Fungal disease, Infection model, Early warning, Climate services

## Abstract

Crop fungal diseases constitute a major cause of yield loss. The development of crop disease monitoring and forecasting tools is an important effort to aid farmers in adapting to climate variability and change. Recognizing weather as a main driver of fungal disease outbreaks, this work assesses the climate suitability for wheat blast (*Magnaporthe oryzae* pathotype Triticum, MoT) development in Asian wheat-producing countries. MoT was reported for the first time in Bangladesh in 2016 and could spread to other countries, provided that environmental conditions are suitable to spore development, distribution, and infection. With results from a generic infection model driven by air temperature and humidity, and motivated by the necessity to assess the potential distribution of MoT based on the response to weather drivers only, we quantify potential MoT infection events across Asia for the period 1980–2019. The results show a potential higher incidence of MoT in Bangladesh, Myanmar, and some areas of India, where the number of potential infection (NPI) events averaged up to 15 during wheat heading. Interannual trends show an increase in NPI over those three countries, which in turns show their higher interannual variability. Cold/dry conditions in countries such as Afghanistan and Pakistan appear to render them unlikely candidates for MoT establishment. The relationship between seasonal climate anomalies and NPI suggests a greater association with relative humidity than with temperature. These results could help to focus future efforts to develop management strategies where weather conditions are conducive for the establishment of MoT.

**Supplementary Information:**

The online version contains supplementary material available at 10.1007/s00484-022-02352-9.

## Introduction

The occurrence of crop diseases caused by fungal pathogens is among the main factors affecting crop yields globally (Figueroa et al. 2018). Although advances in resistant varieties and efficient and environmentally friendly control options are numerous, losses associated with fungal diseases remain very important and, in some cases, devastating (Fisher et al. 2012). The risk of crop disease outbreaks is increasing given the global trade of agricultural commodities, which can increase the exposure of crops to new diseases that have been imported (Bebber et al. 2014). This is the case of wheat blast (MoT) disease caused by the fungus *Magnaporthe oryzae* pathotype *Triticum* (MoT), which evolved and has been present in South America since 1985 (Igarashi et al. 1986). MoT was reported for the first time in South Asia in Bangladesh in 2016 (Malaker et al. 2016; Ceresini et al. 2018), and more recently in Southern Africa in Zambia (Tembo et al. 2020). MoT is considered a potentially devastating fungal disease in countries where it has been historically present, such as Brazil (Igarashi et al. 1986), Bolivia (Barea and Toledo 1996), and Argentina (Perelló et al. 2015), causing periodic and significant yield losses (Cruz et al. 2016; Duveiller et al. 2016). MoT is also an emerging threat to wheat production and food security in countries where wheat is a major staple, such as in Asia (Islam et al. 2020a, b).

In Bangladesh, the first MoT outbreak affected about 15,000 ha of wheat, with an estimated reduction of nearly 30% in production in 2016 (Islam et al. 2020a, b; Yesmin et al. 2020). Although subsequent outbreaks have not been recorded, the disease remains present in Bangladesh with low to moderate severity when detected (Singh et al. 2021). The impacts of MoT on wheat yields and grain quality can be devastating for susceptible cultivars, but they can vary greatly in response to other factors such as weather conditions, growth stage, or planting date (Cruz and Valent 2017). In this way, when weather conditions are suitable for MoT infection, grain yield losses can range from slight to total (Duveiller et al. 2011; Singh et al. 2021), as it has been reported in South American countries such as Brazil or Bolivia, where yield losses have reached up to 100% (Goulart and Paiva 1992; Barea and Toledo 1996).

Although there are still no official reports of the presence of MoT in others countries than Bangladesh in Asia, studies of climate suitability for MoT have suggested that it may spread to areas with humid and warm climates in neighboring countries such as India or Pakistan (Motaleb et al. 2018a). Research has also suggested that Ethiopia (Duveiller et al. 2011) and the USA (Cruz et al. 2016) may be risk-prone. In these regions, both seed-born and air-born spore propagation, suitable weather conditions, and disease susceptible cultivars can act synergistically to increase the risk of disease outbreak, potentially threatening food security (Ceresini et al. 2018). These risks have motivated a number of efforts to monitor pathogen presence (Fernandes et al. 2021; Islam et al. 2016; Yesmin et al. 2020), and to develop management strategies including resistant varieties (Hossain et al. 2019), chemical and nonchemical control methods (Singh et al. 2021), and early warning systems (Fernandes et al. 2021; Kim and Choi 2020).

Multiple tools have been developed for the monitoring and forecasting of fungal disease outbreaks based on field observations or empirical and deterministic models combining weather variables to generate early warnings of the potential risk of disease outbreaks (Launay et al. 2014). Considering MoT in Asia, studies of its potential spread have been carried out using monthly climate statistics (e.g., Motaleb et al. 2018a) or limited temporal and spatial domains (e.g., Kim and Choi 2020). No large-scale, continental, and high-resolution assessments have conversely been carried out in Asia. However, given increasing availability of environmental data and computing capacities, the use of simulation models to diagnose and forecast favorable conditions for the development of crop diseases has grown in importance (Donatelli et al. 2017).

Diagnosis and applications vary from regional assessments of climate suitability (Bebber et al. 2017), sensitivity analysis to environmental drivers and parameterizations (Bregaglio et al. 2012), and future projections in risks associated with climate change (Bregaglio et al. 2013). The latter suggested that the suitable conditions for the establishment of fungal diseases can be well captured by models forced by climate variables (e.g., atmospheric humidity and temperature), provided that parameters are adequately set for a specific disease (Bregaglio et al. 2012; Bregaglio and Donatelli 2015). In the case of MoT, Fernandes et al. (2017) developed a wheat blast–specific model aiming at implementing an early warning system for Brazil, which was applied and evaluated at the local level using a single-location approach and then extended to Bangladesh (Fernandes et al. 2021). The need for decision-making tools for farmers from other wheat-growing regions in Asia has been emphasized later (Singh et al. 2020), given the potential risk for the range of disease expansion (Islam et al. 2020a, b). In this context, the aim of this work is to provide a large-scale and long-term assessment of the climate suitability for MoT development over wheat-growing areas of Asia in terms of mean historical (1980–2019) weather conditions and interannual variability, based on the analysis of the results obtained from high-resolution meteorological data and a generic infection model. The results from this work, which represent an estimate of the potential pressure that can be exerted by MoT driven by background meteorological conditions, can contribute to the understanding of the spatial patterns in suitable weather conditions for MoT and their main large-scale drivers, and can potentially provide guidance for future efforts and regional prioritization in the development of early warning systems based on weather monitoring and forecasting.

## Data and methods

### Study area

Eight Asian countries were identified based on the extent of wheat cultivation and consumption and the recent emergence of wheat blast disease in 2016 in Bangladesh, which are summarized in Table 1 for 2019. In alphabetic order, these include Afghanistan, Bangladesh, Bhutan, China, India, Myanmar, Nepal, and Pakistan. In these countries, winter wheat is planted in the autumn, with a long vegetative stage during the dry season in winter, and the reproductive stage generally occurring with the onset of the spring. In addition, spring wheat is cultivated in areas with mild winters such as in India, and at elevation in the Himalayas, where wheat is sown in autumn and harvested after the winter without vernalization, though land area devoted to spring wheat is limited in South Asia (Curtis 2002; Krupnik et al. 2021). For this reason, this study focuses on winter wheat as the predominant crop. Wheat is a major staple food in Afghanistan and Pakistan, with a total production of 4.9 and 24.3 Mt (million tonnes) over 2.3 and 8.7 Mha (million ha) in 2019, respectively (Fig. S1; FAOSTAT 2021). Wheat consumption has been increasing progressively in India, Bhutan, Myanmar, and Bangladesh, becoming the second most important staple food after rice (Motaleb et al. 2018b). China is the world’s largest wheat producer, with 133.5 Mt grown on 23.7 Mha in 2019; India is the second largest producer, growing 103.6 Mt in 2019 on 29.3 Mha (FAOSTAT 2021). Bangladesh conversely is a net importer of wheat, producing 1 Mt over 0.33 Mha in 2019, cultivated exclusively during the winter after monsoon season rice fields are drained. In Nepal, wheat is grown in the low-lying *Terai* (up to 500 m above sea level) and in the Himalayan mid-hills (Morris et al. 1994; Krupnik et al. 2021), with a total of 2 Mt produced from 0.7 Mha in 2019. In Myanmar, more than 90% of the wheat is found in the hilly Sagaing and Shan states (USDA, 2019), with a production of 110,000 tonnes from 59,000 ha in 2019. In Bhutan, wheat is also produced at elevation, reaching 1319 tonnes from 1004 ha in 2019 (Tshewang et al. 2017).

**Table 1:** Main wheat production statistics for the eight Asian countries considered in this study for the year 2019. Values in brackets correspond to the slope of the linear fit of the corresponding statistics for the period 1961 through 2019. Data from FAOSTAT 2021

Country	Production (tonnes)	Area harvested (ha)	Average yield (tonnes/ha)
Afghanistan	4,890,000 (39,767)	2,334,000 (− 372)	2.095 (0.02)
Bangladesh	1,016,811 (24,511)	330,348 (8360)	3.078 (0.04)
Bhutan	1319 (− 18)	1004 (− 68)	1.314 (0.02)
China	133,596,300 (2,079,597)	23,730,000 (− 37,782)	5.630 (0.09)
India	103,596,230 (1,612,311)	29,318,790 (297,338)	3.533 (0.05)
Myanmar	110,663 (2125)	58,866 (289)	1.880 (0.02)
Nepal	2,005,665 (34,027)	703,992 (12,368)	2.849 (0.03)
Pakistan	24,348,983 (412,302)	8,677,730 (73,567)	2.806 (0.04)

### Modeling potential wheat blast infections

#### Model description

The generic infection model developed by Magarey et al. (2005) was used to assess the climate suitability of MoT infections. This model has been previously applied for large-scale studies of fungal disease infections given the biological significance of its parameterizations and simple implementation (Bregaglio et al. 2012, 2013). The model considers both the effect of hourly air temperature and plant surface wetness (or relative humidity) duration on the development response of a generic fungal pathogen by using two functions describing its sensitivity to air temperature and humidity. The model uses the air temperature response function proposed by Yan and Hunt (1999), which combines a set of pathogen’s cardinal temperatures to estimate the shape of the response as:

1 $$f\left(T\right)=\left(\frac{{T}_{\mathrm{max}}-T}{{T}_{\mathrm{max}}-{T}_{\mathrm{opt}}}\right){\left(\frac{T-{T}_{\mathrm{min}}}{{T}_{\mathrm{opt}}-{T}_{\mathrm{min}}}\right)}^{\left({T}_{\mathrm{opt}}-{T}_{\mathrm{min}}\right)/\left({T}_{\mathrm{max}}-{T}_{\mathrm{opt}}\right)},$$

where *f*(*T*) (dimensionless, values from 0 to 1) is the temperature response function; *T* (°C) is the hourly air temperature; *T*_min_, *T*_max_, and *T*_opt_ are the minimum, maximum, and optimum temperatures for infection, respectively. These cardinal temperatures were taken from Cruz et al. (2016), who suggested the following values for MoT: *T*_min_ = 10 °C, *T*_max_ = 32 °C, and *T*_opt_ = 27.5 °C. As an example, Fig. S2 shows the resulting shape of *f*(*T*), where, following a slow response, exponential increasing response to temperature is observed between *T*_min_ and around 20 °C, which turns from almost linear to a decreasing-rate increment until *T*_opt_, to then drops rapidly until *f*(*T*) = 0 at *T*_max_. The air temperature response *f*(*T*) is subsequently scaled to the wetness duration requirement for infection according to the following relationship:

2 $$W\left(T\right)=\left\{\begin{array}{c}\frac{{WD}_{\mathrm{min}}}{f(T)}, if \frac{{WD}_{\mathrm{min}}}{f(T)}<{WD}_{\mathrm{max}}\\ 0, elsewhere\end{array}\right.,$$

where *W*(*t*) (dimensionless, values from 0 to 1) corresponds to the wetness response function, and *WD*_min_ and *WD*_max_ (hours), taken as 12 and 24, respectively (Cruz et al. 2016), are the minimum and maximum leaf wetness duration requirement for infection, respectively. Therefore, when the infection models use hourly forcing data, it is necessary to account for the number of hours that may interrupt a wet period without terminating the infection process, as Magarey et al. (2005) explained. For this, the model considers the impact of critical dry periods through the parameter *D*50 that is calculated as:

3 $${W}_{\mathrm{sum}}=\left\{\begin{array}{c}{W}_{1}+{W}_{2}, if D\le D50\\ {W}_{1}, {W}_{2}, elsewhere\end{array}\right.,$$

where *W*_sum_ is the sum of the surface wetting periods and *W*_1_ and *W*_2_ indicate two wet periods separated by a dry period (*D*, in hours). As in Magarey et al. (2005), *D*50 is defined as the duration of a dry period at relative humidity < 95% that will result in a 50% reduction in disease compared with a continuous wetness period. Therefore, if *D* > *D*50, the model considers the two wet periods as separate wetting events. When the plant surfaces are wet and *f*(*T*) > 0, the model assumes that inoculant is present in the environment and adds a cohort of spores. Infection events are triggered when the value of *W*_sum_ ranges between *WD*_min_ and *WD*_max_ (Bregaglio et al. 2012). Although the values of the *D*50 parameter were gathered by Magarey et al. (2005) for a number of species of fungal diseases, *D*50 has not yet been calibrated for MoT. We however included a value of *D*50 of 4, which was used by Bregaglio et al. (2013) for the assessment of potential infections of *Pyricularia oryzae*, a MoT anamorph, in Europe (Martínez et al. 2019). The above set of equations were solved for the wheat heading period, which was estimated using a phenological model based on thermal time accumulation, as presented below.

#### Infection model forcing

Multiple global gridded climate products are currently available, which can be potentially used to model and diagnose crop diseases. However, meteorological information must be provided at appropriate temporal and spatial scales, given the behavior of crop pathogens. Among the meteorological variables most used for crop disease modeling are air temperature, precipitation, relative humidity, and leaf wetness (Donatelli et al. 2017). More complex and highly demanding in computer resources, transport-based Lagrangian models require wind speed and direction to calculate fungal spores’ trajectories and deposition (Meyer et al. 2017).

Most global gridded climate products are provided at daily time-steps as the higher temporal resolution, which may be limiting for the simulation of crop diseases. Although there are methods to statistically disaggregate daily time series to hourly values via empirical models or weather generators (Bregaglio et al. 2010), their accuracy can be limited by the available historical data and their implementation can be difficult when it comes to large datasets. This study used hourly data from the last generation European Centre for Medium-Range Weather Forecasts (ECMWF) ERA5 global atmospheric reanalysis as meteorological observations to force the infection model. This product is provided at an hourly time scale with a horizontal resolution of 0.25° × 0.25° (~ 31 km), covering the period 1979 to present for single (surface) and multiple vertical levels (Hersbach et al. 2020). ERA5 is generated using a 4D-Var data assimilation scheme to optimally combine outputs from the ECMWF Integrated Forecasting System with satellite and ground observations. We utilized the hourly ERA5 air and dewpoint temperature at surface level (2 m height), and rainfall data for the period from January 1980 through December 2019. Relative humidity (RH) for the infection model was calculated using the widely used equation involving actual and saturated vapor pressure, which are obtained from dewpoint (*T*_*d*_) temperature and actual air temperature (*T*), respectively (Allen et al. 1998):

4 $${e}_{a}=0.611\times {\text{exp}}^{\left(\frac{17.27\times {T}_{d}}{237.3+{T}_{d}}\right)}$$

5 $${e}_{s}=0.611\times {\text{exp}}^{\left(\frac{17.27\times T}{237.3+T}\right)}$$

6 $$RH=100\times \frac{{e}_{a}}{{e}_{s}}$$

with *e*_*a*_ and *e*_*s*_ are expressed in kPa, and temperatures in °C. Maps of the seasonal climatology of these variables are provided in Fig. S3.

#### Representing wheat distribution and phenology

The spatial distribution of wheat area was represented using the Spatial Production Allocation Model SPAM 2010 v1.0 global crop production data product developed by the International Food Policy Research Institute (IFPRI) (Wood-Sichra et al. 2016; International Food Policy Research Institute (IFPRI) 2019). This product provides statistics on crop production by merging sub-national statistics, satellite-derived land cover, environmental crop suitability, population, cropping systems, and markets, among other variables. The operational product is generated after the crop production data derived from the above-mentioned information is aggregated into a regular grid of spatial resolution of around 10 km × 10 km using a cross-entropy method (You and Wood, 2006). In this work, the original data grid was bilinearly interpolated to the 0.25° × 0.25° climate forcing resolution and then converted into a binary mask (Fig. S4).

MoT infections were estimated for the phenological period from heading to the end of the reproductive phase (maturity). The starting and ending dates of this susceptible period were calculated using crop growth modeling and global climate products. Thus, the spatially explicit critical dates necessary for bounding the modeling time window are sowing date, emergence, beginning of the heading stage, and beginning of physiological maturity. After representing the spatial distribution of wheat, the key phenological dates were stated. First, winter wheat sowing dates were obtained from the interpolated Crop Calendar Dataset of Sacks et al.’s (2010) product, which provides 5′ × 5′ spatial resolution global dates of crop sowing and harvest dates representative of the year 2000. Here, the original resolution dataset was bilinearly aggregated to match the 0.25° × 0.25° ERA5 resolution (Fig. S4).

**Fig. 1:**
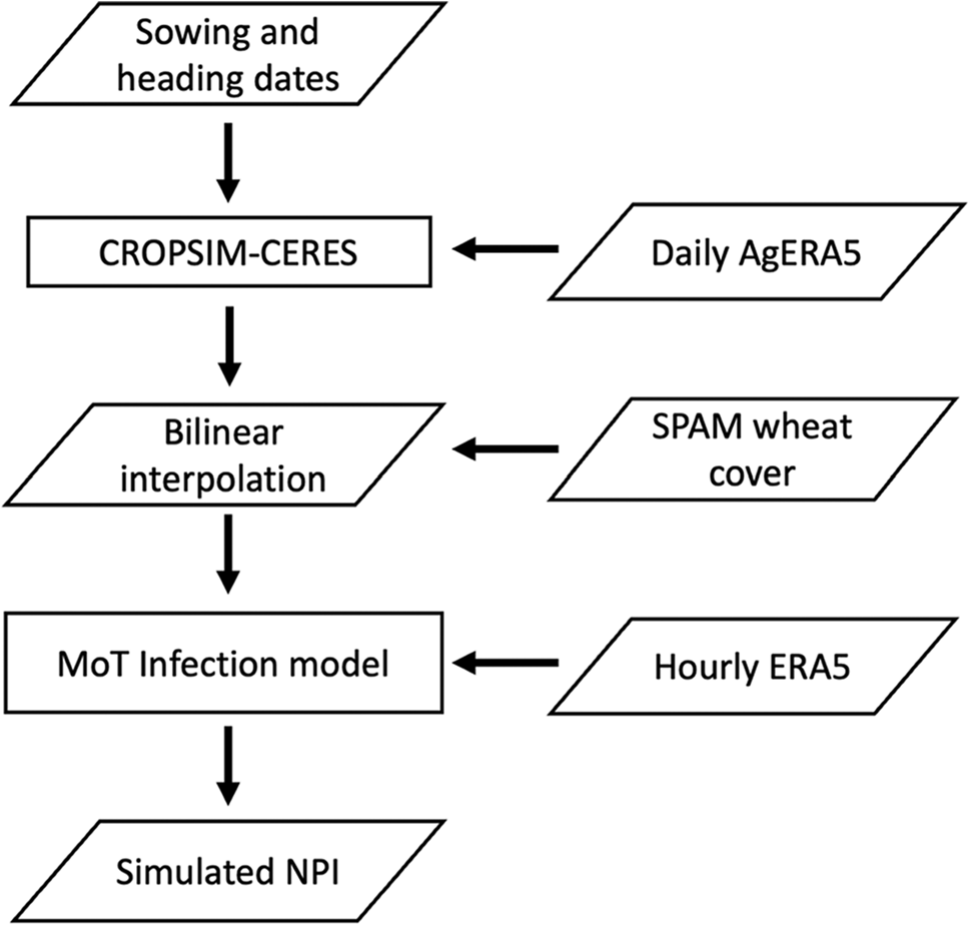
Flow diagram of the modeling approach for number of potential infections (NPI) of MoT. See text for acronyms and product names

The wheat heading period was estimated using point-based simulations with the CSM-CROPSIM-CERES-wheat model, embedded in the Decision Support System for Agrotechnology Transfer (DSSAT) v.4.6 (Jones et al. 2003). CROPSIM-CERES simulates wheat phenology according to the Zadoks stages (Zadoks et al. 1974) as a function of growing degree day accumulation and accounting for environmental stresses, vernalization, and photoperiod effects. Simulations were performed over a set of 163 wheat-growing locations belonging to the International Wheat Improvement Network (IWIN; Reynolds et al. 2017) for the period 1979 through 2019 (Fig. S5). The meteorological forcing (air temperature, solar radiation, rainfall, relative humidity, wind speed) was performed using ECMWF’s AgERA5 product (Copernicus Climate Change Service (C3S), 2019), a statistically downscaled (0.1° × 0.1°) daily version of ERA5. Global soil profiles from the HC27 product (Koo and Dimes 2010) were used to provide soil physical and chemical properties to CROPSIM-CERES. Genetic coefficients necessary for wheat simulations were set based on the cultivar distribution over the International Maize and Wheat Improvement Center’s (CIMMYT’s) wheat mega-environments, which correspond to homogeneous agroecological zones for wheat cultivation (Pequeno et al. 2021). A comparison between CROPSIM-CERES simulated number of days from sowing to anthesis and IWIN observations showed a normalized root mean square error of 7.6% (data not shown). Finally, using sowing dates from Sacks et al. (2010), simulated dates of anthesis and maturity were obtained for every location, and the heading date was assumed to occur 10 days before anthesis. Both heading and maturity dates were bilinearly interpolated to 0.25° × 0.25° working spatial resolution of the SPAM product (Fig. S5). A flow diagram schematically describing the main steps of the modeling approach is shown in Fig. 1.

### Analysis

Data analysis involved three analytical steps. The first step focused on the quantification of the average and interannual variability (1980–2019) in the weather-driven number of potential infections (NPI) of MoT summarized for all selected countries, except for Bhutan, where model results showed non-suitable climate conditions for wheat blast development. The interannual trends (slope of the linear fit) in NPI were also evaluated, and their statistical significance was assessed using the non-parametrical Mann–Kendall test (Kendall, 1955) at a confidence level of 0.05. In the second step, the covariability between NPI and climate variables was assessed. This was performed by computing the Pearson correlation coefficient between pairs of detrended time series of NPI and air temperature, relative humidity, and rainfall anomalies. Lastly, a composite analysis of anomalies of the above-presented climate variables was performed for the years of highest MoT incidence predicted by the model, taking the upper quartile (75th percentile) of the NPI time series. Anomalies were obtained by removing the corresponding long-term average.

## Results

### Mean patterns and interannual variability of number of potential MoT infections

**Fig. 2:**
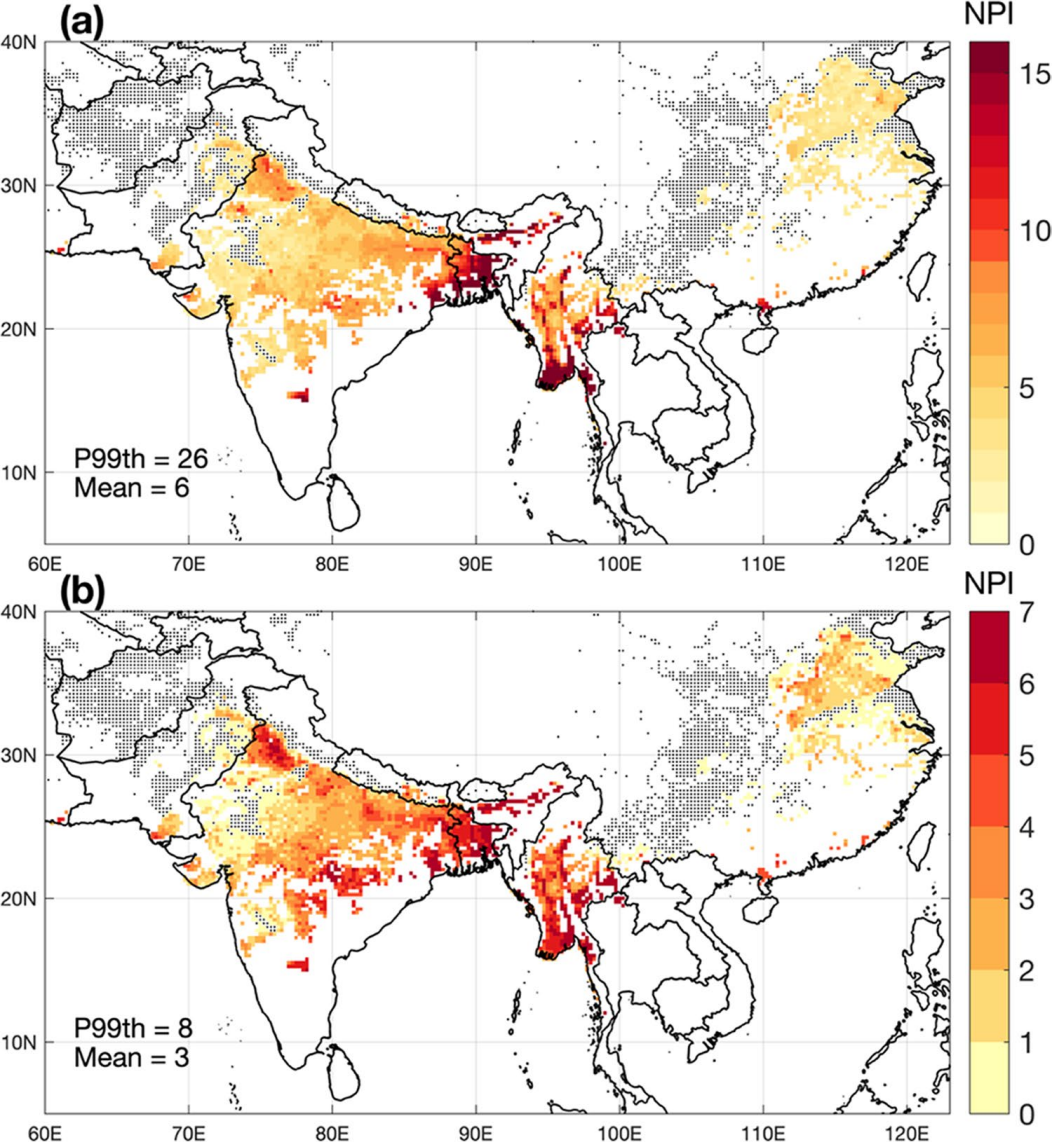
Maps of **a** mean and **b** interannual variability represented by the standard deviation (1980–2019) of the number of *Magnaporthe oryzae* pathotype Triticum (MoT) potential infections (NPI) in Asia. Black dots represent grid cells with presence of wheat but where the climate appears to not be suitable for MoT outbreaks. P99th is the 99% percentile

**Fig. 3:**
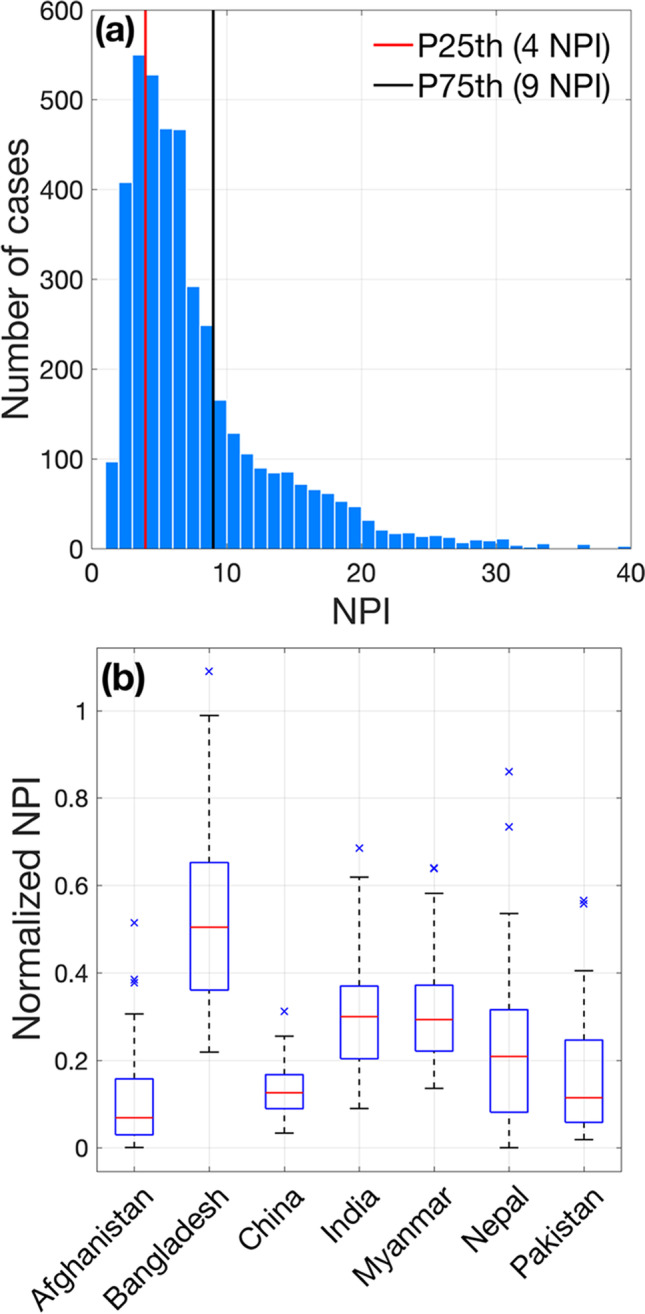
**a** Histogram of multi-year number of potential MOT infections (NPI) in Asia; P25% and P75% are the corresponding percentiles. **b** Boxplots of interannual distribution of NPI. In **b**, the red central mark shows the median and the box edges are the 25th and 75th percentiles; dashed lines extend to the most extreme values not considered outliers, and outliers are plotted individually (× signs)

Figure 2a shows the map of interannual mean total seasonal NPI in Asia. The mean seasonal NPI is 7.5 (median of 6) and interquartile range from 4 to 9 (Fig. 3a). In Fig. 2a, while 57.6% of SPAM wheat grid cells present suitable conditions for MoT, 6.7% of them present favorable conditions during all 39 years studied. The map shows a spatial distribution of NPI indicating higher climate suitability for MoT development over areas near the ocean over the southern fraction of the domain, such as in Bangladesh, some areas of West Bengal and Bihar India, and in Myanmar. The model suggests that MoT can also establish over large areas of central India, Myanmar, and China, though at lower NPI levels. Conversely, other wheat-producing regions have air temperature and humidity ranges that would not represent favorable conditions for MoT outbreaks, including most areas in Afghanistan, Pakistan, and central China. Figure 2b shows the interannual variability (standard deviation) of potential infections in Asia, where a strong interannual variability is observed in areas of higher incidence (Bangladesh, Myanmar), but also a southward increase in potential infection risks in India. Similarly, interannual variability of NPI shows a wider range over the areas of higher MoT potential incidence (e.g., Bangladesh). The southward increasing pattern in India and Myanmar suggests that the pressure of the disease could be much higher than the average conditions during more favorable years for its development, so long as sufficiently susceptible wheat cultivars are grown and alternative hosts maintain inoculum outside the wheat-growing season. The distributions of total multi-year NPI cases and NPI normalized by the corresponding infected area and aggregated by country are presented in Fig. 3a and b, respectively. There is a considerable variation in the mean and spread of the distribution of normalized NPI across countries. However, it is clear that Bangladesh is the country with the highest relative potential MoT pressure associated with climate, followed by India and Myanmar, which present similar disease risk scenarios, and then in Nepal, China, Pakistan, and Afghanistan.

**Fig. 4:**
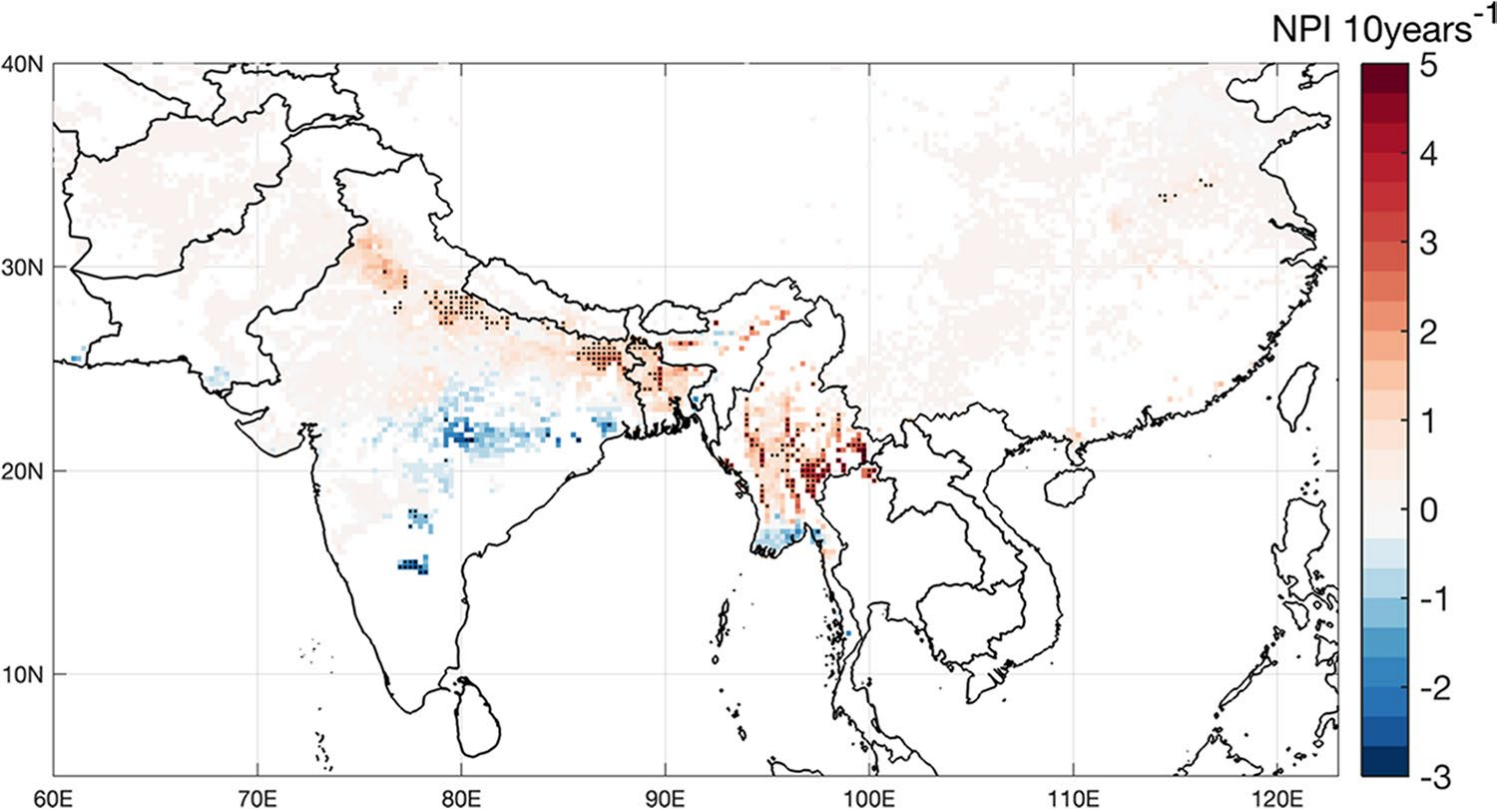
Map of interannual trends (1980–2019) in NPI over Asia. Black dots represent areas where linear trends are statistically significant (*α* = 0.05) according to the Mann–Kendall test

The long-term interannual trends in seasonal total NPI are displayed in Fig. 4, including their statistical significance. Our model outputs suggest generally increasing trends in NPI that concentrate over areas of higher MoT pressure shown in Fig. 2a, although only a small fraction is statistically significant according to the Mann–Kendall test. Positive trends are dominant in Bangladesh, central Myanmar, and over portions of the Indo-Gangetic Plains (IGP) of India. Decreasing trends are observed further south over warmer areas of India and in Myanmar’s delta, where recent temperature trends may be above the maximum MoT development temperature in the model (IPCC 2021).

### Seasonal climate anomalies and number of potential MoT infections

**Fig. 5:**
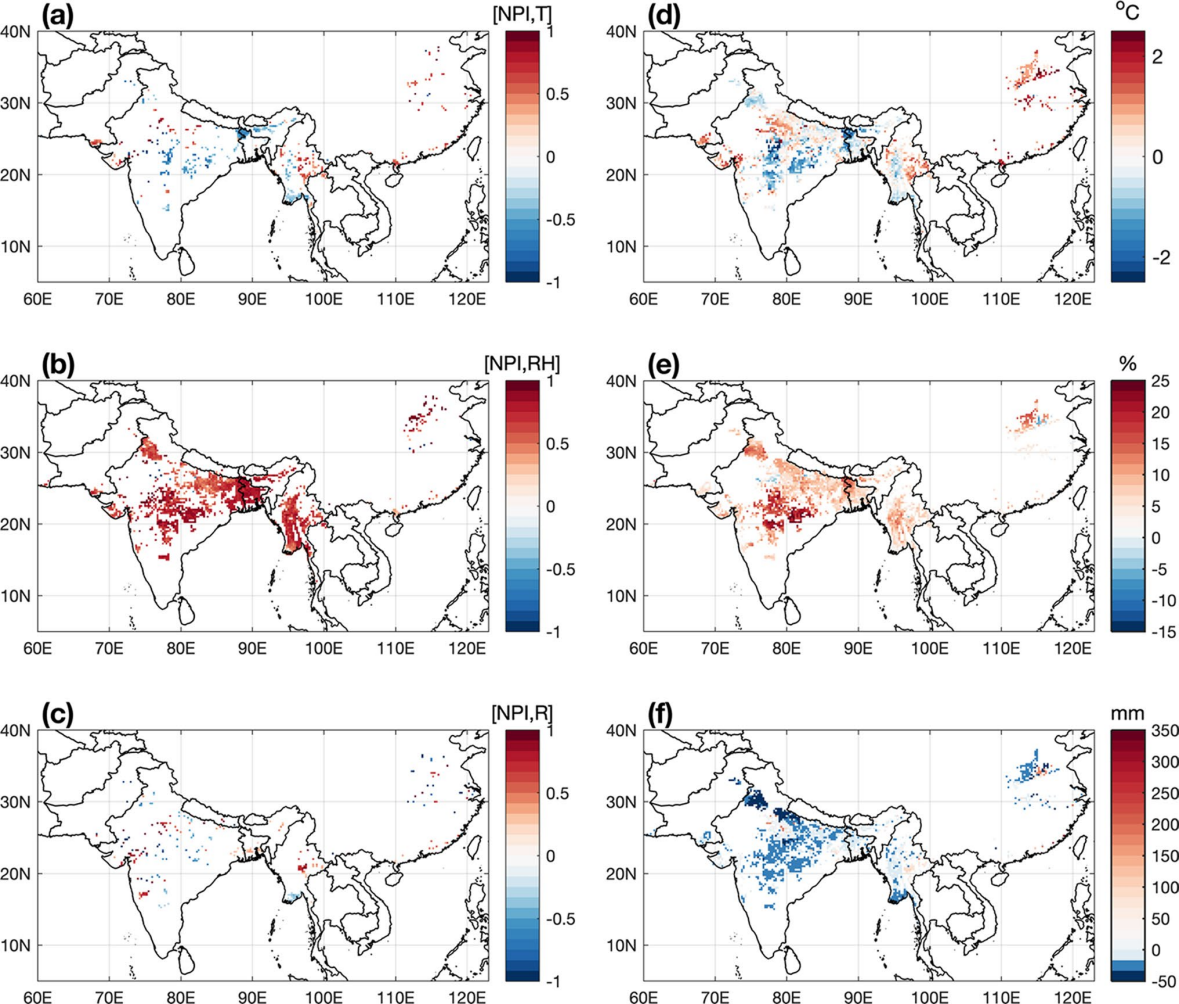
**a**–**c** Maps of local Pearson correlation coefficient between number of potential infections (NPI) and **a** mean temperature (T), **b** relative humidity (RH), and **c** total rainfall (R). Only significant correlations at the 10% level are displayed. **d**–**f** Composites of seasonal **a** mean air temperature, **b** relative humidity, and **c** total rainfall associated with the upper quartile of NPI. Only grid cells exceeding the 95% confidence interval are displayed in **d**–**f**

The relationship between NPI and seasonal anomalies (from wheat heading to maturity) of air temperature (T), relative humidity (RH), and total rainfall (R) is described in Fig. 5a–c, which show the correlation coefficient calculated between NPI and these variables. The correlation map of NPI and T shows that most of the grid points with suitable climate conditions for MoT described in Fig. 2 do not present statistically significant correlations. This is likely due to the scaling used in the temperature response function (Eq. 1), which implies a non-linear relationship between temperature and NPI. However, a small area in northern Bangladesh with relatively high MoT pressure (Fig. 2) has significant negative correlations. This area exhibits high temperatures during the wheat heading period (Fig. S6), which may imply a higher frequency of hours with the temperature above the range of suitability considered in the model, indicative of reduced infection risk potential. On the other hand, the correlation between NPI and RH (Fig. 5b) is much more apparent than with temperature; this is indicative of the importance of RH conditions for the potential development of the disease. In this case, strong positive correlations are observed over Bangladesh, Myanmar, and some areas of India, which correspond to those of higher MoT pressure (Figs. 2 and 3). A very weak correlation between NPI and total rainfall is observed in Fig. 5c for the whole geographical domain. Since the calculation period falls in general within the dry season (Fig. S6), our models suggest that precipitation may not be a significant determining factor of the incidence of MoT at the scales of the present work, as other factors associated with atmospheric water vapor transport might be more relevant (Ahmed et al. 2020). However, the 2016 outbreak of MoT in Bangladesh has been associated with strong storm events during the dry season (Singh et al. 2021), which is not captured by a correlation-based analysis.

The maps of mean composite anomalies of T, RH, and R calculated for the years of the highest infection events, represented by the upper quartile of NPI, are displayed in Fig. 5d–f. Figure 5d shows an area of negative temperature anomalies in northern Bangladesh that was already observed with high interannual correlations in Fig. 5a. In general, both India and Myanmar present a pattern of anomalies that are not very clear, although they in general follow what is observed in terms of correlations (Fig. 5a). Conversely, RH shows a spatial distribution (Fig. 5e) that appears to be consistent with the interannual correlations (Fig. 5b), where anomalously high seasonal NPI is associated with positive anomalies in RH, appearing again as a variable with high discriminatory power for modeled NPI. Figure 5f shows that high incidence of MoT is likely to be associated with negative precipitation anomalies. The latter suggests that, despite the null correlation between both variables, drier-than-normal winters may tend to be more favorable for MoT outbreaks.

## Discussion

### Climate suitability for MoT in Asia

Assuming the presence of inoculum and susceptible cultivars, the primary goal of this study was to provide a general and objective overview of the background climate conditions for the development of MoT over a region where wheat cultivation is important for food security (Yonar et al. 2021), and whose agricultural landscape is described as being highly exposed to the shocks associated with weather and climate variability (Amarnath et al. 2017). At the time of writing, MoT has only been officially reported in Bangladesh (Malaker et al. 2016), though unofficial reports of the disease have also been published in the popular media in eastern India, prompting the temporary banning of wheat cultivation in some areas (cf. Islam et al. 2020a, b); our modeling efforts also suggest high disease pressure risks, though not always with significant and positive interannual trends, potentially backing the consistent but spatially variable incidence and severity of MoT observed in this country between 2017 and present (CSISA 2021). The reasons for the variable nature of infections observed in Bangladesh and South Asia remain unclear; though recognizing weather conditions as a major driver of fungal disease outbreaks (Bregaglio et al. 2012; Juroszek et al. 2019), improved knowledge regarding the environmental suitability for the establishment of MoT can aid in anticipating the development of disease management strategies. These include but are not limited to the deployment of new resistant varieties, cultural control methods, and the use of early warning systems in wheat regions where the disease is a potential threat for food security.

The results highlight the importance of spatial variability in the climate suitability for the establishment of MoT in Asia, with a higher potential observed in Bangladesh, Myanmar, and some areas of India, where low elevation, sea proximity, or regional low-level circulation can favor factors such as atmospheric water transport (Ahmed et al. 2020). Using a methodologically similar approach, a similar spatial pattern in the suitability of rice leaf blast (*Magnaporthe oryzae* pathotype Oryzae) driven by summer weather over North India was described by Viswanath et al. (2017) for India. At the same time, regions that appear to have higher potential risks for infection in our model are also associated with higher interannual variability. This appears to reflect in literature on MoT from South America (Fernandes et al. 2017) and observations in Bangladesh (CSISA 2021) that the disease is irregularly periodic, increasing in incidence and severity only during years of higher favorable conditions. On the other hand, we also observed increasing NPI risks in northwestern India. This result could potentially be associated with irrigation, which is intensively applied to wheat on over 80% of the land area devoted to rice–wheat rotations in northwestern India (Hussain et al. 2003; Jain et al. 2017; Ram et al. 2013;), which could contribute to land surface cooling during the pre- and post-monsoon period (Mishra et al. 2020). Intensified use of irrigation on the other hand has also increased evaporation, increasing actual water vapor pressure (Tuinenburg et al. 2014), which can determine an increase in relative humidity creating conditions that are more suitable for MoT development (Bregaglio et al. 2012). Additionally, our scenarios suggest that high incidence of MoT is likely to be associated with negative precipitation anomalies. The latter could be associated with the regulatory effect of rainfall on air temperature, which affects relative humidity during a period of the year where precipitation events are sporadic. However, further analysis is necessary to validate this hypothesis.

On the other hand, results suggest that wheat-producing regions with low temperature and humidity in Afghanistan, Pakistan, or some areas of India are unlikely to be at significant risk for MoT outbreaks, as climatic regime appears to be out of the range for the disease development (Magarey et al. 2005; Cruz et al. 2016). According to the observed relationship between interannual variability in NPI and the selected climate variables, a clear association between anomalies of RH and NPI was observed, which is explained by the structure of the infection model. This observation confirms those of Kim and Choi (2020) and Fernandes et al. 2017) that suggested that this variable could be potentially used for the development of seasonal early warning systems. Indeed, recent efforts to develop weather-based early warning systems in Bangladesh and Brazil (e.g., Fernandes et al. 2021; http://beattheblastews.net/) rely largely on humidity and temperature as driving variables. Nevertheless, the association between NPI and climate anomalies seems to be clearer when using a composite approach, which could open the possibility of generating probabilistic seasonal forecasts of favorable conditions for MoT outbreaks. The latter could be further explored using indices from large-scale drivers (El Niño/La Niña) and suitable lead times. In addition, although most of the area of the geographical domain studied did not exhibit statistically significant trends, areas that exhibit show positive trends in NPI that could increase in response to projected climate change scenarios, which should be addressed in future studies.

### Limitations and uncertainties

The approach used in this study considers the combination of multiple sources of secondary information and modeling (cropping calendars, phenology, potential infections, etc.). This in turn implies multiple sources of uncertainty and limitations that should be considered in order to improve the understanding of the conditions conducive to the development of crop diseases, including future projections in climate. For instance, the generic infection model considers the moment when favorable weather conditions for the development of MoT are fulfilled to declare an outbreak. However, other complex disease-host interaction processes could determine the successful establishment of the disease, which are not considered in the model, which also assumes that inoculant is present in the environments being studied (e.g., Bregaglio et al. 2012, 2021). Additionally, other relevant variables that have been considered in other similar works could be included. For instance, the “wash-off” effect of spores from spikes by rainfall above a specific intensity has been considered by Fernandes et al. (2017), which can be relevant over more where significant rains occur during the heading wheat period. Another source of uncertainty are the model parameters, and specifically the *D*50 parameter used in Eq. 3, which was extracted from similar fungal species (*Puccinia* sp. and *Bipolaris* sp.) in rice and wheat. Although this can lead to errors, Bregaglio et al. (2012) found that results are not very sensitive to the values of *D*50 for other fungal diseases. We however conducted a simple sensitivity analysis, S7, that was performed using a set of values of *D*50, from “sensitive” to “insensitive” to dry interruptions according to Magarey et al. (2005), presented in Fig. S7, which suggests that the calculated NPI are not very sensitive to variations in *D*50 values. In any case, a global sensitivity and uncertainty quantification analysis would provide a better understanding of the model structure and sensitivity to parameters and threshold values (Bregaglio et al. 2012), which is, however, out of the scope of this work.

In addition, and in spite of using phenology dates that are comparable to other works (e.g., Liu et al. 2020), the use of fixed planting and heading/maturity dates may represent a source of error during, for instance, anomalously warm/cold years, in which the phenological stages can be accelerated/delayed. The latter may represent a limitation when developing early warning systems based on seasonal climatic forecasts, which currently provide information on a monthly or longer scale. Moreover, we relied on a single, though comprehensive data source for planting dates from the interpolated Crop Calendar Dataset of Sacks et al. (2010). Although widely used, this dataset may have inaccuracies with observed planting dates, which can in turn affect phenological development. For example, this dataset shows quite late wheat sowing dates into December in the north western IGP, and specifically in the Indian states of Haryana and Punjab (Fig. S4). These locations however tend to be associated with earlier planting than in the eastern IGP (Lobell et al. 2013; Jain et al. 2017). The reasons for the lack of congruence between the Sacks et al. (2010) dataset and observations are not clear, although future studies should query the relationship between crop establishment dates and disease incidence, in an effort to identify if and how MoT risks could be mitigated through manipulation of sowing dates. Remote sensing–based regional sowing date estimations based on the seasonality of satellite time series such TIMESAT (Jönsson and Eklundh, 2004) could help to generate global products of key phenological stages. Similarly, although the SPAM dataset (Wood-Sichra et al. 2016; International Food Policy Research Institute (IFPRI) 2019) is widely used (e.g., Joglekar et al. 2019; Yu et al. 2020), it is partially based on administrative report data for 2005 and has not been thoroughly ground-truthed and as such there may be spatial over- and under-estimation of wheat cultivation area. For example, Myanmar has a declining trend and less than 100,000 ha of wheat (FAOSTAT 2021; USDA PS&D 2022), while the SPAM product suggests a cultivation area of around 85,500 ha, and a more southern distribution of cultivation than other sources suggest (USDA PS&D 2022). Future researchers may therefore consider making use of satellite-derived estimates for wheat phenology to complement this data source, for example, using methods described by Jain et al. (2017). Yet despite these potential inconsistencies, our model outputs still provide a useful indication of the potential for MoT infection risks, and can therefore be used to help in crop planning and zoning, in addition to integrated pest management efforts, although care should be exercised when interpreting our results.

## Conclusions

The sudden, unexpected arrival of wheat blast disease in Bangladesh in 2016 underscores the risk associated with this disease. Although formally reported in Bangladesh at the time of writing, there is a lack of clarity on the potential distribution of the MoT species and its effect on wheat cultivation throughout the Asian continent. Our results suggest a differential suitability for the development of MoT—and a large interannual variation in some key wheat-producing areas—across Asia. The contrasting potential risk of MoT between Bangladesh, Myanmar, and some states within India, with infection events averaging up to 15 during the wheat spike, and limited risks in Afghanistan and Pakistan, and in central China, could allow focusing efforts to increase the resilience and preparation of farmers for potential future biotic shocks. Importantly, our results also highlight a stronger association between relative humidity and MoT infection than with temperature regime. Accordingly, future improvements should further investigate if and how relative humidity can be used to simplify data requirements and modeling efforts. New research should also focus on including more complex pathogen-plant interaction processes, dynamic wheat phenology, source-sink relationships, and wind dispersal patterns, higher resolution climate forcing for historical and future assessments. Although still preliminary in nature, our results nonetheless may aid in the development or refinement of early warning systems and agricultural climate services associated with MoT and similar diseases.

## Supplementary Information

Below is the link to the electronic supplementary material.

Supplementary file1 (DOCX 4521 KB)
