# Supplementary material for: Variable climate suitability for wheat blast (Magnaporthe oryzae pathotype Triticum) in Asia: results from a continental-scale modeling approach
Source: Int J Biometeorol. 2022 Aug 22;66(11):2237–49. doi: 10.1007/s00484-022-02352-9 (PMC9640415; doi:10.1007/s00484-022-02352-9)
Supplement: Supplementary file 1 — Supplementary file1 (DOCX 4521 KB) [file 484_2022_2352_MOESM1_ESM.docx]

**Variable climate suitability for wheat blast (*Magnaporthe oryzae* pathotype Triticum) in Asia:**

**Results from a continental-scale modeling approach**

Carlo Montes^1^^[[1]](#footnote-1)^, Sk. Ghulam Hussain^2^, Timothy J. Krupnik^2^

^1^International Maize and Wheat Improvement Center (CIMMYT), Texcoco, Mexico

^2^ International Maize and Wheat Improvement Center (CIMMYT), Dhaka, Bangladesh

**Supplementary information**


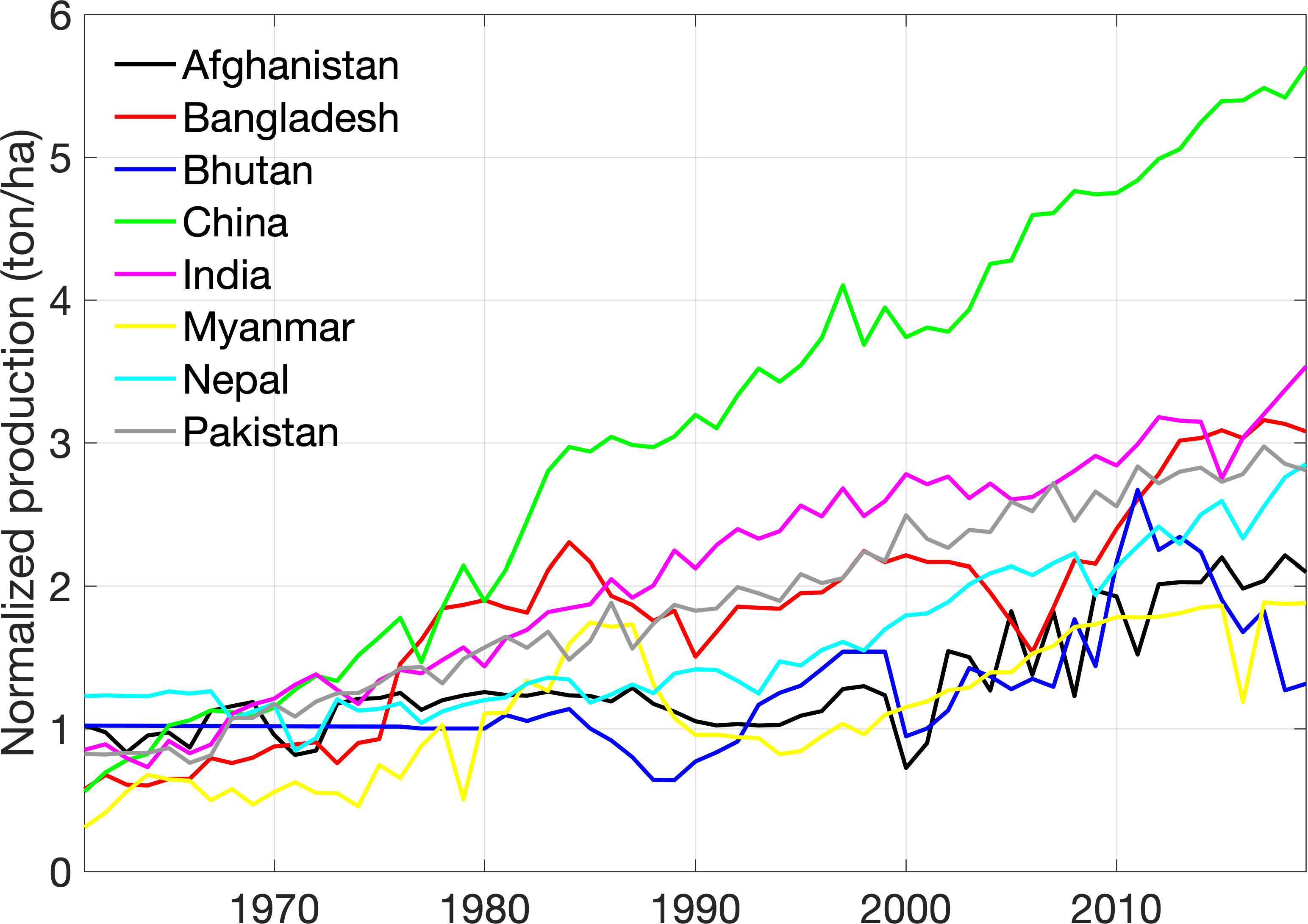


Figure S1. Time series (1961-2019) of wheat production in the countries considered in this study (data from FAOSTAT 2021).


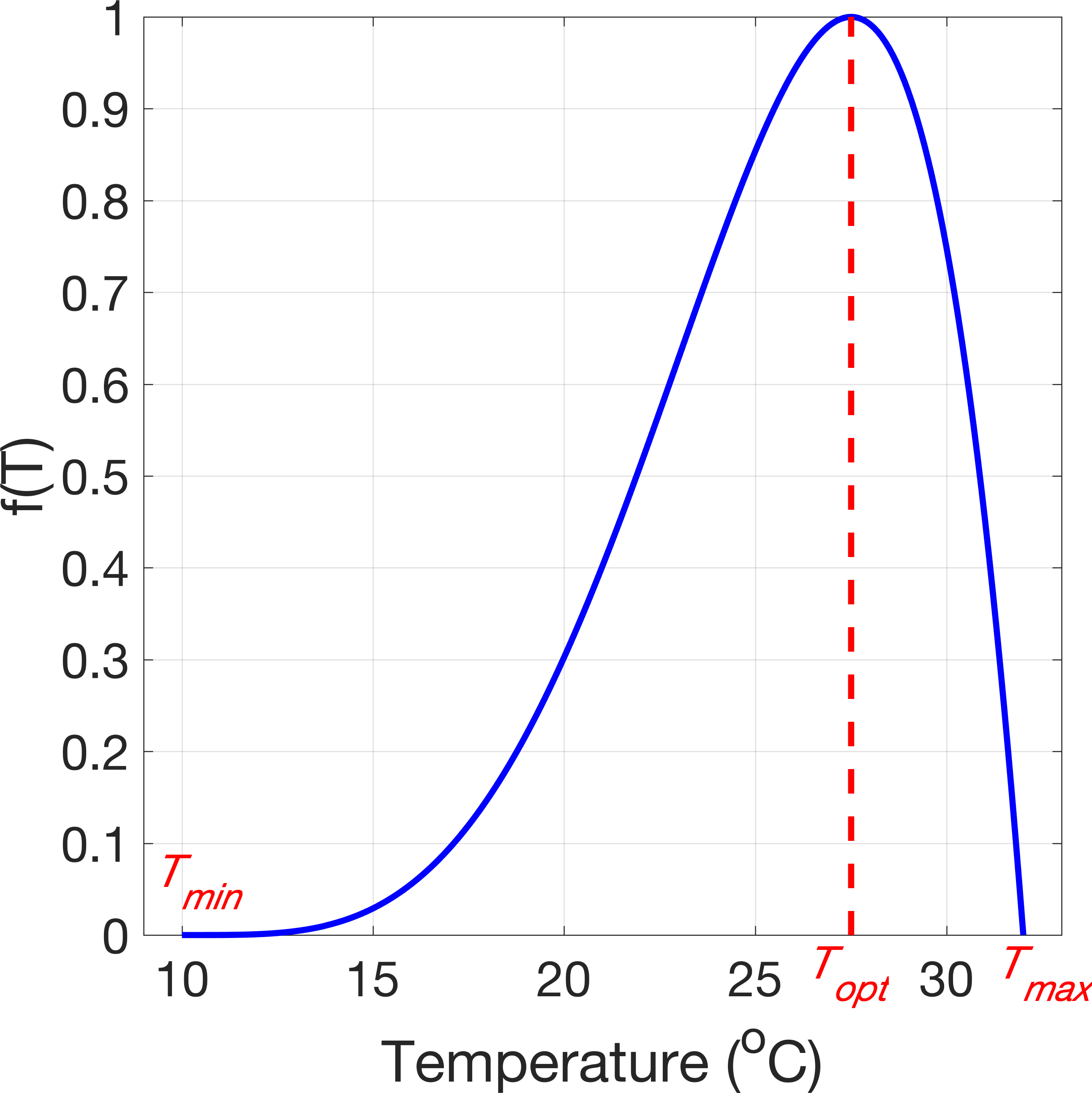


Figure S2. The shape of temperature response curve obtained by Equation (1) using parameters for wheat blast (explained the text).


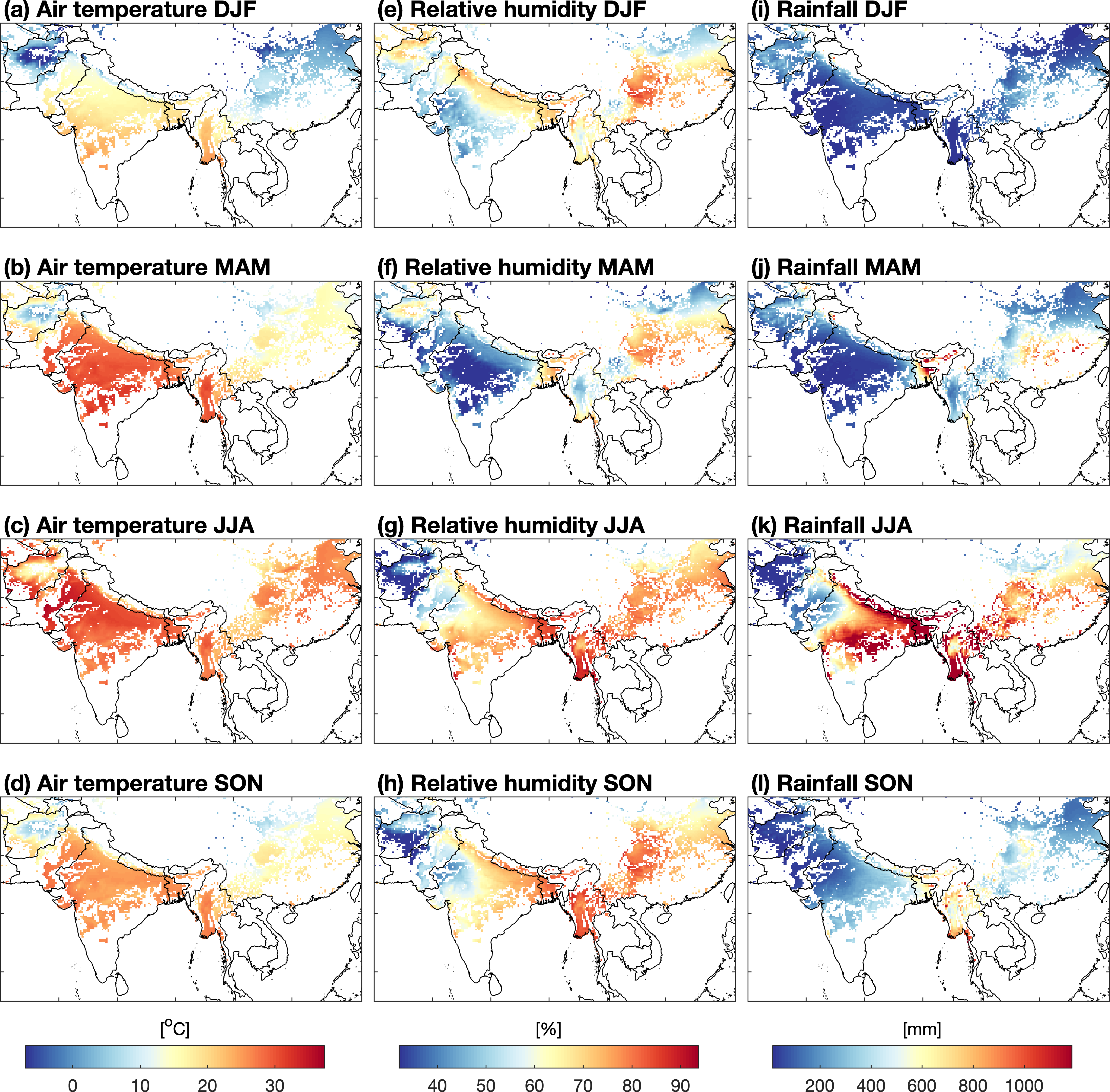


Figure S3. Climatology (1980-2019) of seasonal average mean of daily (a)-(d) air temperature, (e)-(h) relative humidity, and (i)-(l) total rainfall from ERA5. DJF, MAM, JJA and SON indicate December, January, and Febuary, March, April, and May, June, July, and August, and September, October, and November, respectively.


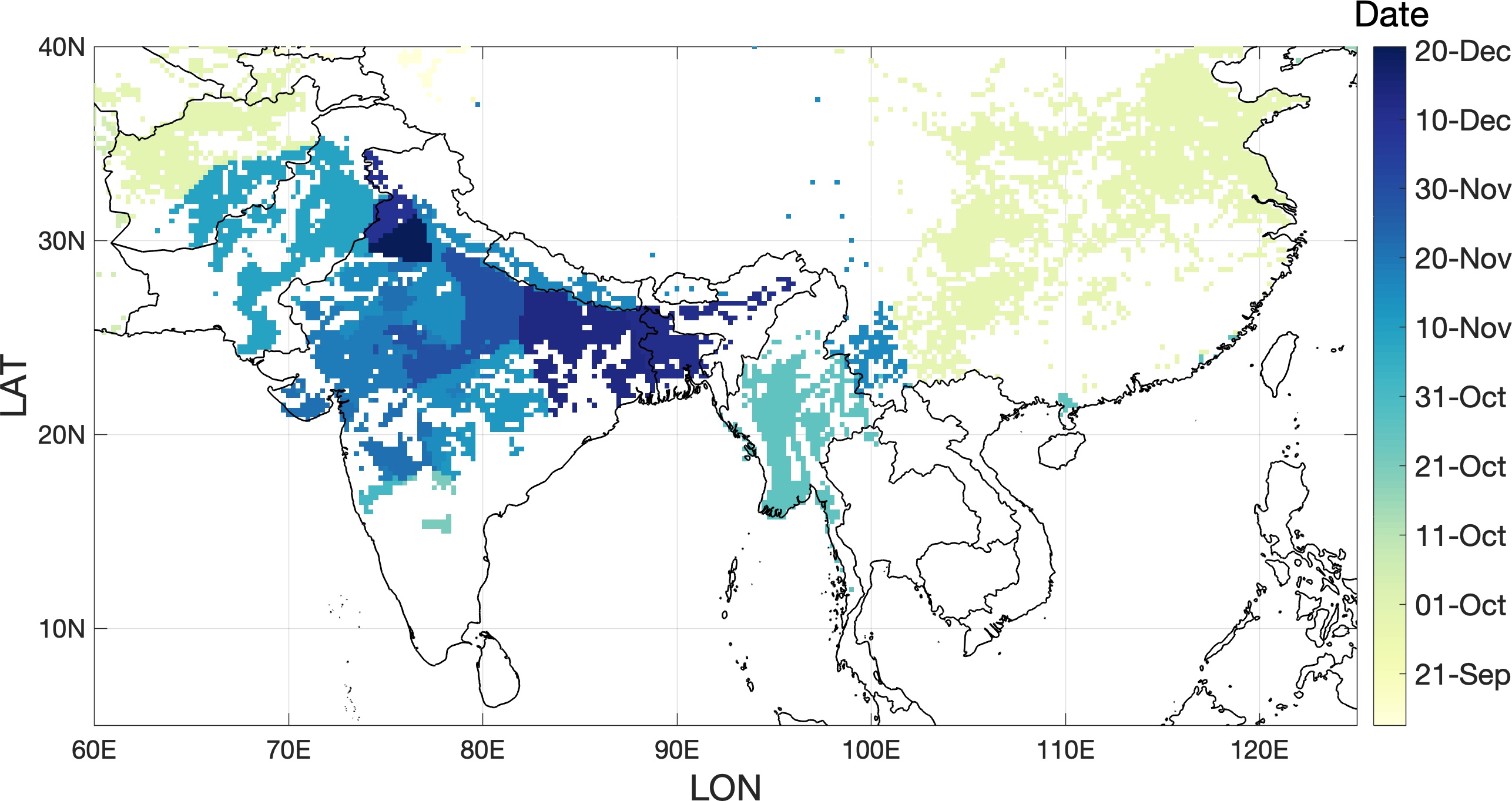


Figure S4. Map of wheat sowing dates for wheat in Asia over grid cells of the Spatial Production Allocation Model wheat area.


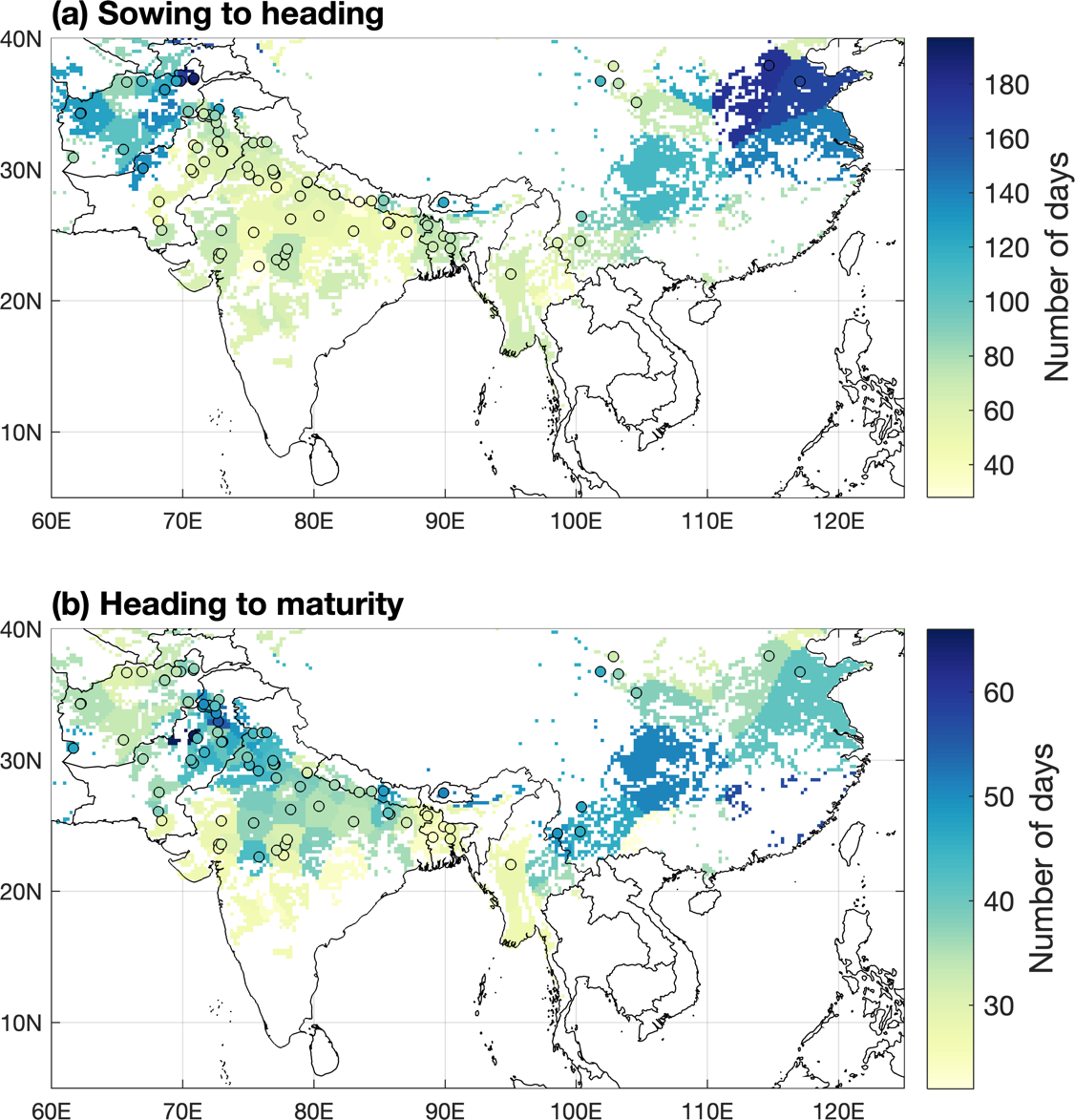


Figure S5. Map of interpolated (colored areas) number of days between (a) sowing and heading date and (b) heading to maturity. Circles correspond to International Wheat Improvement Network locations of CROPSIM-CERES simulations (*n* = 163).


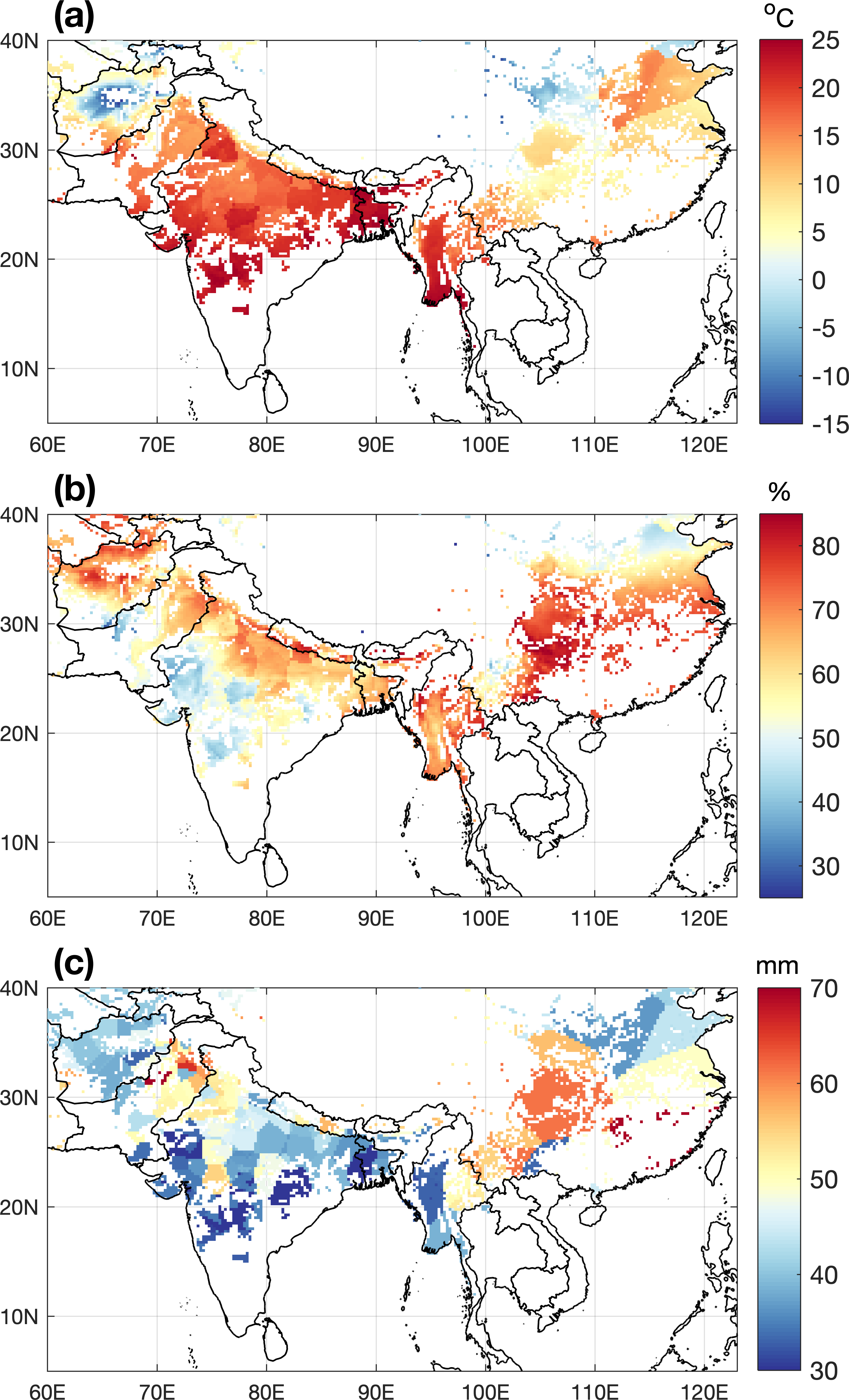


Figure S6. Climatology (1980-2019) of (a) air temperature, (b) relative humidity, and (c) total rainfall during the wheat heading period.


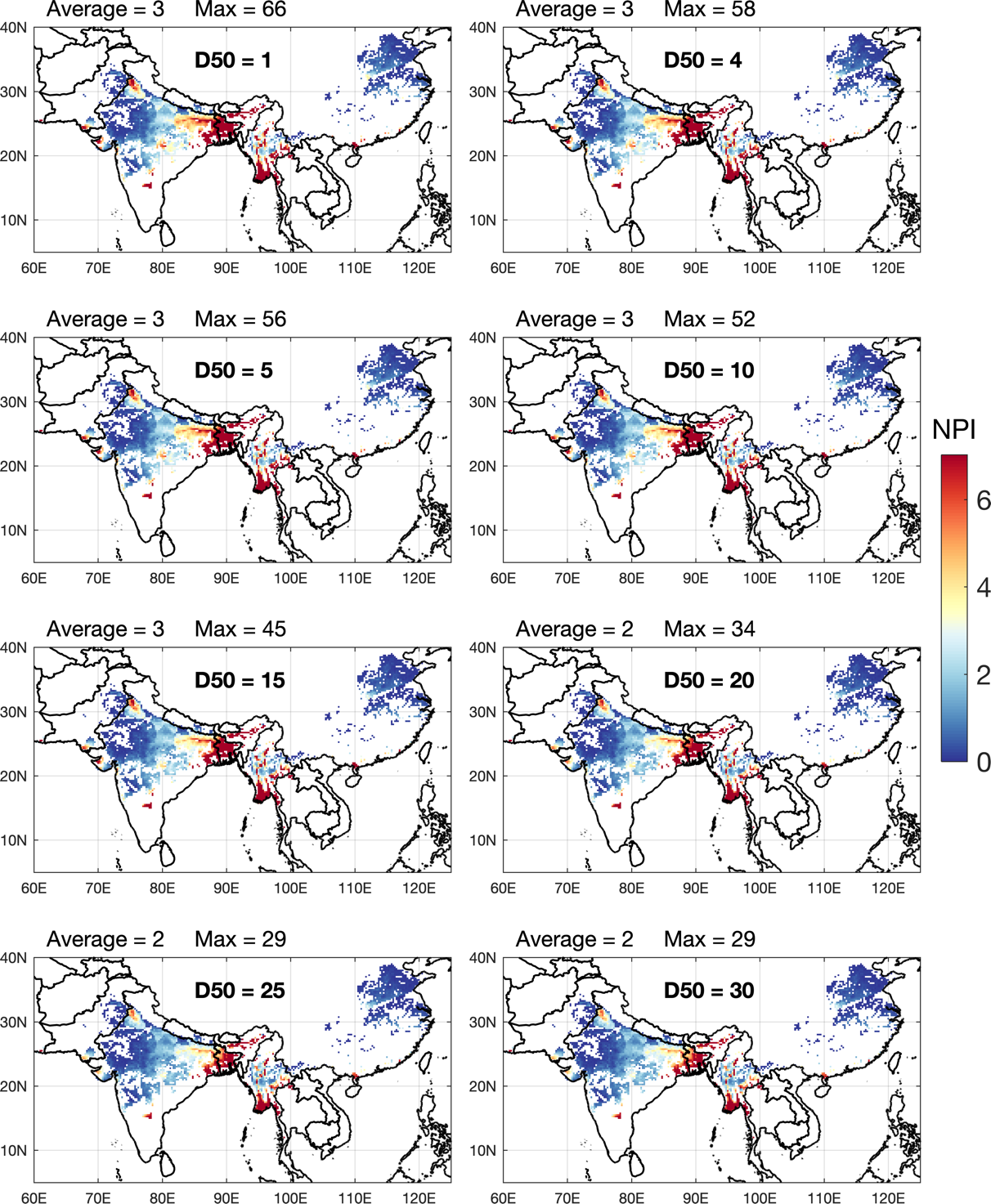


Figure S7. Sensitivity analysis showing average and maximum number of potential wheat blast infections (NPI) for different values of the D50 parameter.

1. Corresponding author: c.montes@cgiar.org [↑](#footnote-ref-1)
